# CAREGIVERS’ PERCEIVED IMPORTANCE IN SOCIAL ACTIVITIES EXACERBATES THE EFFECT OF SOCIAL RESTRICTIONS ON DEPRESSION

**DOI:** 10.1093/geroni/igae098.1510

**Published:** 2024-12-31

**Authors:** Yuanchang Zhao, Mengzhao Yan, Jessica Kelley

**Affiliations:** Case Western Reserve University, Cleveland, Ohio, United States; University of Southern California, Los Angeles, California, United States; Case Western Reserve University, Cleveland, Ohio, United States

## Abstract

Taking on a family caregiving role can limit time for social activities, even ones that are very important to the caregiver. Despite the well-known benefits of social activity participation, such as mental health and reducing caregiver strain, few studies have examined the potential impact of missing specific types of social activities and their degree of importance for caregivers on their mental health. Guided by the conceptual model of caregiver burden and role theory, we hypothesize that missing specific activities that are important to the caregiver will have a more negative impact on depressive symptoms. We utilize Round 12 National Study of Caregiving (NSOC) data of family caregivers to people aged 65+ (N=2,431) focused on four key social activities: visiting friends and family, attending religious services, participating in group activities, and enjoying outdoor events. Our findings indicate that for each additional activity caregivers miss, they have 54% increased odds of depressive symptoms. Further, missing any activity considered “important” by the caregiver had 70% higher odds of depressive symptoms. Missing outdoor activities increased odds of depressive symptoms by 216%, but missing outdoor activities considered “important” had 234% higher odds of depressive symptoms. In sum, limiting social and recreational activities due to caregiving raises the risk of poor mental health, but missing activities that are highly valued by the caregiver raises that risk even further. These findings underscore the importance of recognizing caregivers’ perceived important activities to better understand and address the mental health challenges they face due to activity restrictions.

